# Structural Interactions between Inhibitor and Substrate Docking Sites Give Insight into Mechanisms of Human PS1 Complexes

**DOI:** 10.1016/j.str.2013.09.018

**Published:** 2014-01-07

**Authors:** Yi Li, Stephen Hsueh-Jeng Lu, Ching-Ju Tsai, Christopher Bohm, Seema Qamar, Roger B. Dodd, William Meadows, Amy Jeon, Adam McLeod, Fusheng Chen, Muriel Arimon, Oksana Berezovska, Bradley T. Hyman, Taisuke Tomita, Takeshi Iwatsubo, Christopher M. Johnson, Lindsay A. Farrer, Gerold Schmitt-Ulms, Paul E. Fraser, Peter H. St George-Hyslop

**Affiliations:** 1 Department of Clinical Neurosciences, Cambridge Institute for Medical Research, University of Cambridge, Cambridge CB2 0XY, UK; 2 Tanz Centre for Research in Neurodegenerative Diseases, and Departments of Medicine, Laboratory Medicine and Pathobiology, and Medical Biophysics, University of Toronto, Toronto, ON M5S 3H2, Canada; 3 Alzheimer Research Unit, MassGeneral Institute for Neurodegenerative Diseases, Massachusetts General Hospital, Charlestown, MA 02129, USA; 4 Department of Neuropathology and Neuroscience, Graduate School of Pharmaceutical Sciences, and Department of Neuropathology, Graduate School of Medicine, The University of Tokyo, Tokyo 113-0033, Japan; 5 Core Research for Evolutional Science and Technology, Japan Science and Technology Agency, Bunkyo-ku, Tokyo 113-0033, Japan; 6 MRC Laboratory of Molecular Biology, Cambridge Biomedical Campus, Francis Crick Avenue, Cambridge CB2 0QH, UK; 7 Departments of Medicine (Biomedical Genetics), Neurology, Ophthalmology, Genetics and Genomics, Biostatistics, and Epidemiology, Boston University School of Medicine, 72 East Concord Street, Boston, MA 02118, USA

## Abstract

Presenilin-mediated endoproteolysis of transmembrane proteins plays a key role in physiological signaling and in the pathogenesis of Alzheimer disease and some cancers. Numerous inhibitors have been found via library screens, but their structural mechanisms remain unknown. We used several biophysical techniques to investigate the structure of human presenilin complexes and the effects of peptidomimetic γ-secretase inhibitors. The complexes are bilobed. The head contains nicastrin ectodomain. The membrane-embedded base has a central channel and a lateral cleft, which may represent the initial substrate docking site. Inhibitor binding induces widespread structural changes, including rotation of the head and closure of the lateral cleft. These changes block substrate access to the catalytic pocket and inhibit the enzyme. Intriguingly, peptide substrate docking has reciprocal effects on the inhibitor binding site. Similar reciprocal shifts may underlie the mechanisms of other inhibitors and of the “lateral gate” through which substrates access to the catalytic site.

## Highlights

• The head contains nicastrin ectodomain and overhangs a solute-accessible cavity in base
• The base has a central channel and a lateral cleft (putative substrate docking site)
• Inhibitors close the cleft and channel and rotate the head, blocking substrate access

## Graphical Abstract

**Figure:**
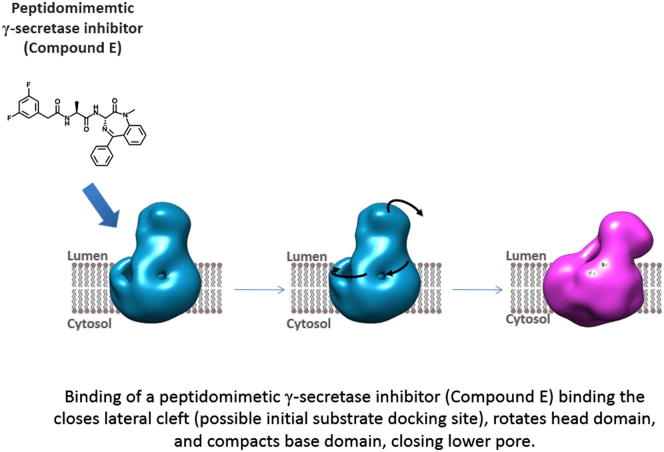

## Teaser

Presenilin complexes mediate proteolysis of transmembrane proteins during physiological signaling and disease. Li et al. describe the architecture of human presenilin complex PS1 and inhibitor-induced structural changes. They propose that similar shifts likely underlie substrate access to the catalytic site.

## Introduction

**Figure 1:**
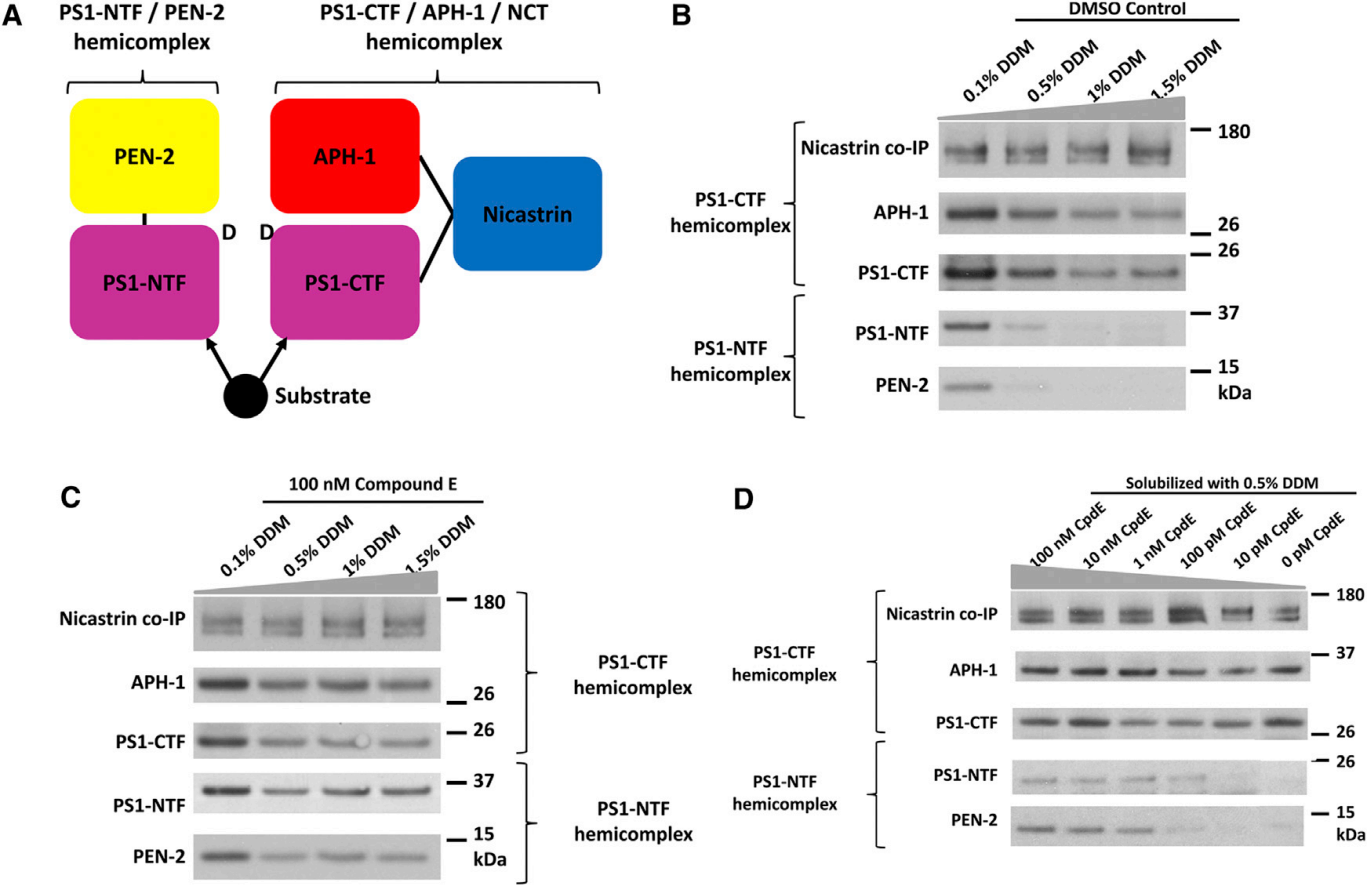
Compound E Binding Induces Conformational Changes in PS1 Complexes that Prevent Detergent-Mediated Dissociation of PS1 Complexes into Hemi-Complexes (A) Cartoon depicting the hemi-complexes. Substrates have been previously shown to bind to both PS1-NTF and PS1-CTF. (B) In 0.1% DDM, nicastrin coimmunoprecipitates all complex components: PS1-NTF, PS1-CTF, aph1, and pen2. With increasing detergent concentration, nicastrin coimmunoprecipitates only aph1 and PS1-CTF. (C) Incubation of complexes with compound E stabilizes complexes across a range of detergent concentrations. (D) The compound E-mediated stabilization of the PS1 complexes is dose dependent.

Presenilin complexes (also known as γ-secretase complexes) are composed of four core component proteins: presenilin 1 (PS1; Sherrington et al., 1995) or presenilin 2 (PS2; Rogaev et al., 1995); anterior pharynx 1 (aph1; Francis et al., 2002, Goutte et al., 2002); presenilin enhancer 2 (pen2; Francis et al., 2002); and nicastrin (Yu et al., 2000) (Figure 1A). A subset of complexes may also contain one or more regulatory proteins (e.g., transmembrane emp24 transport domain-containing protein 10 [Chen et al., 2006] and γ-secretase activating protein [He et al., 2010, St George-Hyslop and Schmitt-Ulms, 2010]). During maturation and activation of the complex, the presenilin holoproteins undergo autocatalytic cleavage to generate N-terminal fragments (PS1-NTFs) and C-terminal fragments (PS1-CTFs; Figure 1A; Thinakaran et al., 1996). The mature presenilin complexes then perform the intramembranous endoproteolysis of several biologically important Type I transmembrane (TM) proteins, including Notch, p75, and the amyloid precursor protein (APP; Haass and Selkoe, 2007). This cleavage is catalyzed by two aspartate residues that are thought to be located in a hydrophilic pocket surrounded by the TM domains of the core complex proteins—one located on TM6 in the PS1-NTF, the other on TM7 in the PS1-CTF (Wolfe et al., 1999).

The recent crystal structure of MCMJR1 (also known as *Methanoculleus marisnigri* presenilin/SSP homolog), a distantly related Archeal homolog of the human PS1 subunit (19.3% sequence identity; PDB code 4HYC; Li et al., 2013), confirmed that the catalytic aspartates are located in a hydrophilic pocket surrounded by the TM domains of the MCMJR1 protein. However, many critical questions still remain about the structural biology of both the Archeon PS1-subunit homolog and the multimeric eukaryotic and presenilin complex. For instance, it has been speculated that substrates may gain access to the active site of the presenilin aspartyl protease family by a “lateral gate” mechanism involving lateral movement of the substrate TM between the TM domains of the protease. In MCMJR1, it has been speculated that this might occur via a lateral cleft between TM6 and TM9 (Li et al., 2013). However, nothing is known about the mechanics of this putative “lateral gate.” Similarly, although functional screens of chemical libraries have yielded numerous small molecule inhibitors and modulators (Wolfe, 2012), the structural mechanisms are unknown for most of these compounds (Fuwa et al., 2007, Kornilova et al., 2005, Ohki et al., 2011, Sato et al., 2008, Tian et al., 2002, Tian et al., 2003, Watanabe et al., 2010).

We reasoned that some of these inhibitors, especially highly potent peptidomimetic inhibitors like compound E, might be used as tools to explore the functional biology of the presenilin complex. Such studies could provide insight into the mechanisms by which noncatalytic site inhibitors work. They could also shed light on both the structural plasticity of the complex and how long-range interactions within the complex might modulate its catalytic activity. We chose to use compound E ((S,S)- 2-[2-(3,5-difluorophenyl)-acetylamino]-N-(1-methyl-2-oxo-5-phenyl-2,3-dihydro-1H-benzo[e][1,4]diazepin-3-yl)-propionamide) for these studies. Compound E is a small molecule (MW = 490.5 Da) whose backbone structure resembles a papride bond. This peptidomimetic inhibitor previously has been shown to bind to a noncatalytic site on PS1-NTF (Fuwa et al., 2007) and to have very powerful γ-secretase inhibitor activity (50% maximal inhibitory concentration of 0.3 nM; Seiffert et al., 2000). The peptidomimetic nature of compound E, together with its potent and specific inhibitory activity, suggested that it likely binds to sites on PS1-NTF that are functionally important in substrate access to the active site of the presenilin complex.

Here, we report the results of experiments applying several complementary methods to investigate the structure of the native human PS1 complex and of the human PS1 complex after the binding of compound E. We show both directly (by negative-stain single-particle electron microscopy [EM]) and indirectly (by biochemical, pharmacological, and intramolecular fluorescent lifetime imaging microscopy—Förster resonance energy transfer [FLIM-FRET] methods) that inhibitor binding induces long-range changes in structure and function of the complex. These changes include rotation of the nicastrin-containing head domain, compaction of the membrane-embedded base domain with closure of the lateral cleft, and functional closure of the initial substrate docking site. We show that there are also reciprocal long-range interactions between the initial substrate docking site and the inhibitor binding site whereby substrate docking opens the inhibitor binding site. Taken together, these observations describe the inhibitory mechanism of compound E. However, our observations also demonstrate that the presenilin complex is structurally dynamic. They show that there are important reciprocal long-range structural interactions occurring between different sites within the complex and that these structural interactions have powerful effects on the catalytic activity of the complex.

## Results

### Compound E Protects PS1 Complex from Detergent-Induced Dissociation

We and others (Fraering et al., 2004) have previously shown that detergents cause a concentration-dependent separation of the presenilin complex into two hemi-complexes (Figures 1A and 1B). One hemi-complex contains pen2 and PS1-NTF (bearing one catalytic aspartate on TM6 and one-half of the initial substrate docking site). The other hemi-complex contains nicastrin, aph1, and PS1-CTF (bearing the other catalytic aspartate on TM7 and the other half of the initial substrate docking site; Figures 1A and 1B). However, binding of compound E caused a dose-dependent resistance to this detergent-induced dissociation of the two hemi-complexes (Figures 1C and 1D). This observation suggested that compound E might induce significant structural rearrangements in the complex that brings the component proteins into closer proximity. Such closer proximity could then promote stronger interactions between the two hemicomplexes, rendering them resistant to detergent-induced separation.

### Effect of Compound E on Intramolecular FRET

**Figure 2:**
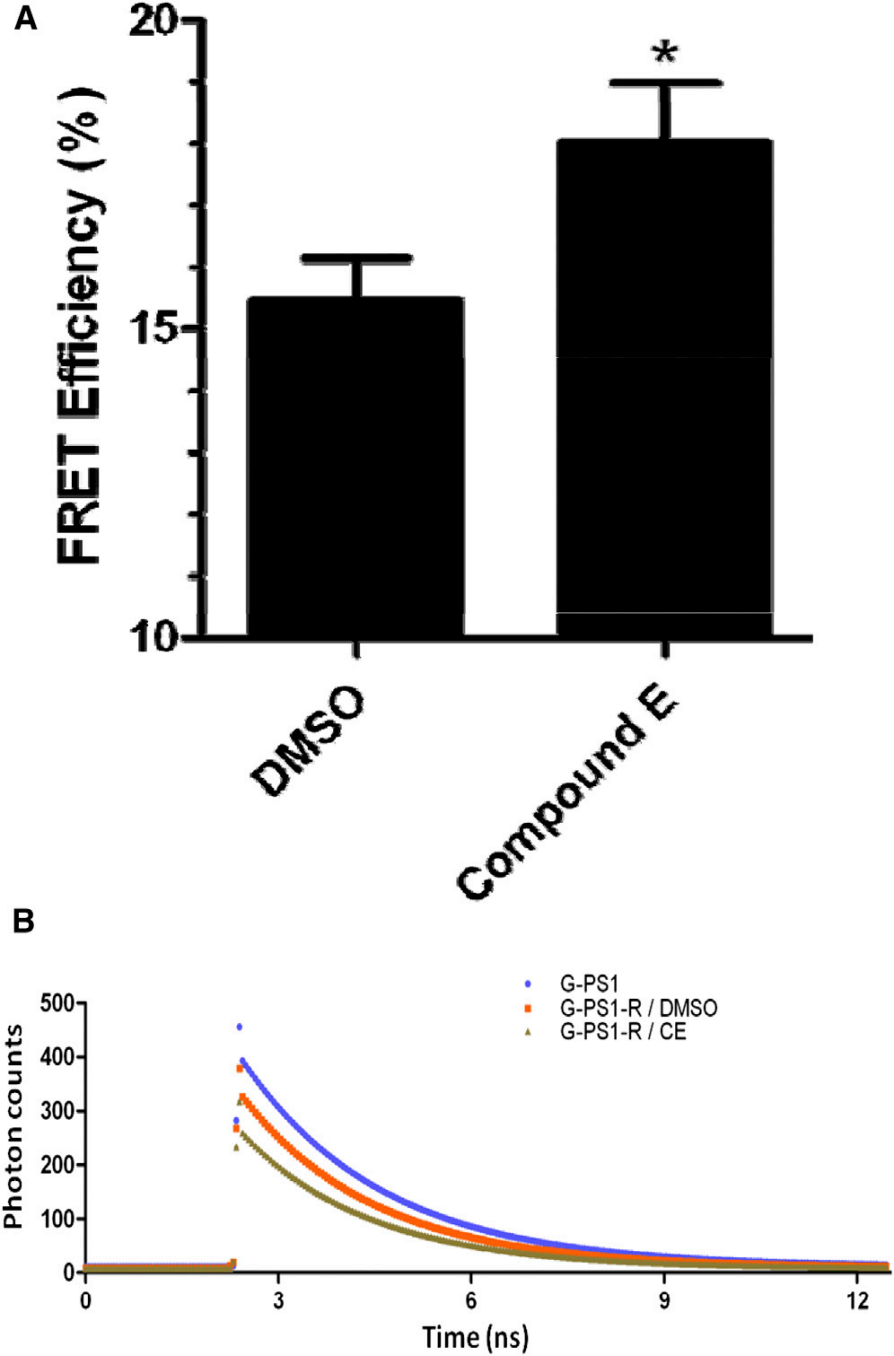
FLIM-FRET Analysis of Purified, Mature, Catalytically Active, GFP- and RFP-Tagged PS1 Complexes Confirms that Compound E Binding Causes Conformational Changes (A) FRET efficiencies of GFP/RFP-tagged PS1 complexes are improved after incubation in 10 μM compound E (DMSO control: 15.46 ± 0.69% mean ± SEM; 10 μM compound E: 18.02 ± 0.95%, n = three independent experiments). ^∗^p ≤ 0.05. (B) Representative fluorescence decay curves for donor GFP fluorescence under each experimental condition. G-PS1 and G-PS1-R decay curves and fluorescence lifetimes were derived from fitting the experimentally observed photon counts by single exponential decay and double exponential decay models, respectively. The relationship of the FRET donor-receiver pair on the two hemi-complexes is displayed in Figure S1A. A western blot demonstrating complete endoproteolysis and maturation of the GFP/RFP-tagged PS1 complexes is displayed in Figure S1B.

To test this hypothesis, we applied intramolecular FLIM-FRET methods on PS1 complexes that were doubly tagged with both GFP at the N terminus and red fluorescent protein (RFP) at codon 351 (Figure S1A available online). The doubly tagged PS1 cDNA was constructed so that, after the physiological endoproteolysis of the PS1 holoprotein, the GFP tag at the N terminus of TM1 would label the <PS1-NTF + pen2> hemi-complex. The RFP tag at the N terminus of TM7 would label the <PS1-CTF + aph1 + nicastrin> hemi-complex (Herl et al., 2006, Uemura et al., 2010). We expressed the GFP-PS1-RFP protein in murine PS1-PS2 double-knockout fibroblasts and purified the resulting mature, catalytically active, doubly-tagged PS1 complexes by wheat germ agglutinin (WGA) chromatography (Figure S1B). The complexes were then subjected to FLIM-FRET analysis in the presence or absence of excess compound E. Native complexes exhibited 15.46 ± 0.69% FRET efficiency. In contrast, complexes with compound E bound had significantly higher FRET efficiencies (18.02 ± 0.95%, p = 0.0321; Figures 2A and 2B). The higher FRET efficiency in compound E-bound complexes supports the notion that binding of compound E induces significant structural changes in the complex that result in a more compact conformation.

### Compound E Has Allosteric Effects on the Initial Substrate Docking Site

**Figure 3:**
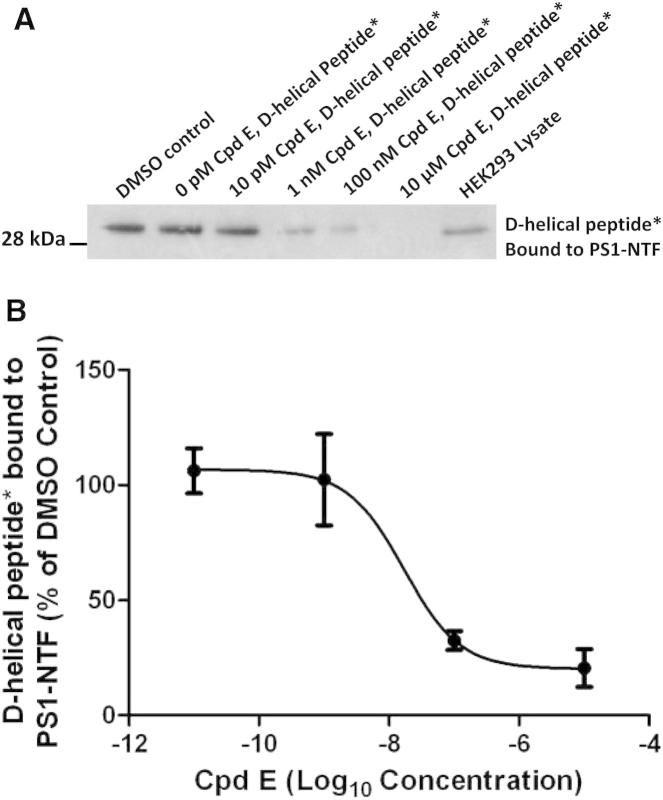
Noncleavable Peptidic D-Helical Substrate Mimic and Compound E Have Reciprocal Allosteric Effects on Each Other’s Binding to PS1 Complexes and Compound E Inhibits Binding of the D-Amino-Acid Helical Substrate to the Initial Substrate Docking Site of PS1 Complexes (A) A representative blot showing progressive inhibition of D-helical photoprobe binding to PS1-NTF in the presence of increasing concentrations of compound E. (B) Quantitative results of four independent experiments expressed as percentage of DMSO control. Error bars are SEM.

**Figure 4:**
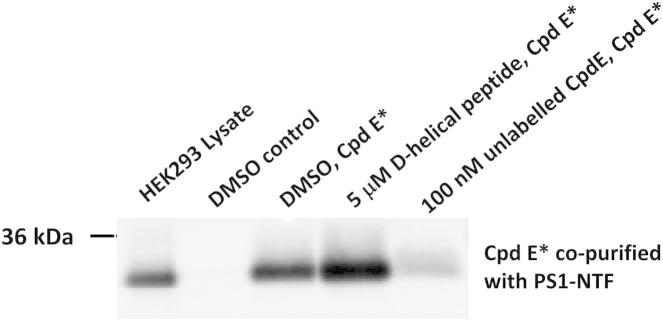
Noncleavable Peptidic D-Helical Substrate Mimic and Compound E Have Reciprocal Allosteric Effects on Each Other’s Binding to PS1 Complexes Preincubation of the PS1 complexes with the D-helical substrate mimic enhanced binding of compound E to the PS1 complex.

The observation that binding of compound E caused significant conformational changes in the PS1 complex raised the possibility that these changes might also have long-range (allosteric) effects on other functional domains of the presenilin complex (e.g., the initial substrate docking site; Figure 1A). To address this question, we monitored the docking of a noncleavable substrate in the presence of varying concentrations of compound E (10 pM–10 μM; Fuwa et al., 2007, Kornilova et al., 2005, Watanabe et al., 2010). Compared with native PS1 complexes, compound E-bound PS1 complexes showed dose-dependent reductions in binding of the substrate (Figures 3A and 3B). Intriguingly, in the reciprocal experiment, preincubation of the PS1 complex with the noncleavable substrate resulted in increased binding of labeled compound E (Figure 4). This enhanced binding of labeled compound E was specific because it could be blocked with excess of unlabeled compound E. Taken together, these experimental results demonstrate a hitherto unrecognized reciprocal long-range structural interaction within the complex. Compound E binding to PS1-NTF “closes” the initial substrate docking site at the interface of PS1-NTF and PS1-CTF. Conversely, substrate binding “opens” the compound E inhibitor binding site.

To investigate the structural basis of these allosteric interactions, we used negative-stain EM to compare the three-dimensional (3D) structures of native and compound E-bound complexes.

Human PS1 complexes were captured (in the presence or absence of compound E) by tagging the N terminus of PS1 with a tandem affinity purification (TAP) tag and expressing the TAP-tagged PS1 subunit at near-physiological levels in human embryonic kidney 293 (HEK293) cells. The tagged complexes were solubilized in digitonin and purified by three-step affinity chromatography (Figure S2). The resulting complexes were pure, mono-dispersed, structurally intact, and enzymatically active (Figures S3A–S3C). The masses of the PS1 protein complex and of the associated detergent molecules were determined using size exclusion chromatography with multiangle light scattering (SEC-MALS). The estimated mass of the catalytically active PS1 complex was 174 kDa, suggesting that the PS1 complex has 1:1:1:1 stoichiometry in solution. The mass distribution evaluated across the main protein peak was constant, indicating that there was a single major, highly monodispersed species (Figure S4). This result is of note because a few prior studies have suggested that the PS1 complex may exist as a dimer and because the crystal structure of the Archeon homolog of the eukaryotic PS1 subunit can be interpreted to suggest that it exists as a tetramer (Li et al., 2013).

### Negative-Stain EM

**Figure 5:**
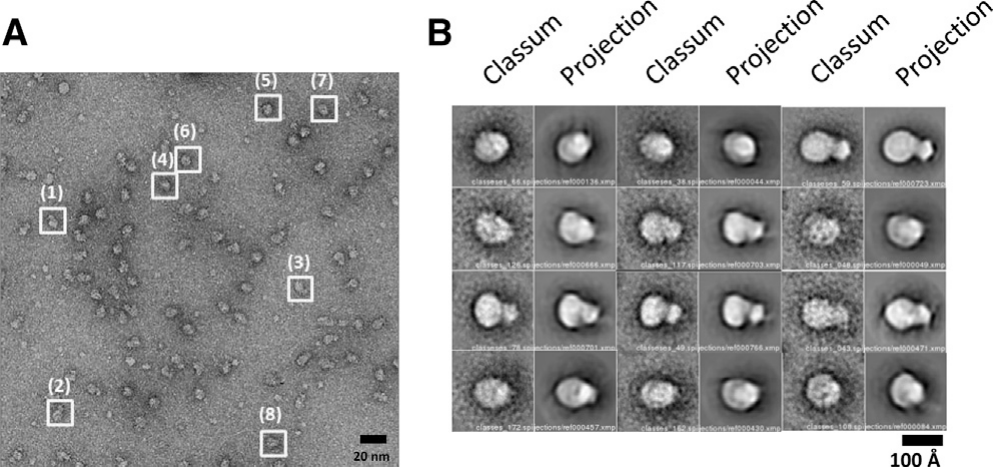
Raw Particles and 3D Model Validation of Human PS1 Complex (A) CCD image of native PS1 complexes. Representative particle shapes are highlighted by white boxes; boxes 1–3, bilobed shapes; boxes 4–8, round or oval shapes; boxes 6–8 show suggestive central cavities. Scale bar, 20 nm. (B) Classums of native PS1 particles compared with 2D projections of the final model for native PS1 complexes. The classums and corresponding 2D projections are highly similar in size, shape, and internal density distribution. Bilobed, oval, and round shapes are seen that are similar to the raw particle images. Central cavities in the base domain are apparent in most classum/2D-projection pairs. Scale bar, 100 Å. Supplemental information is available, including a detailed flowchart of the complex purification algorithm (Figure S2), silver-stained SDS-PAGE gel showing the presence of all complex components and blue native PAGE showing their monodispersity and catalytic activity that can be inhibited by compound E (Figure S3), mass analysis of the complex using size exclusion chromatography with SEC-MALS (Figure S4), detailed flowchart of the model-building algorithm (Figure S5), and additional classum images (Figure S6).

Native and compound E-bound PS1 complexes were then negatively stained and imaged by EM. The resultant images revealed individual particles adopting different orientations with characteristic asymmetric, round, oval, or bilobed shapes of ∼100 Å in diameter (Figure 5A; Table 1; Figure S6).

**Table 1:** Summary of the Untilted and RCT EM Images, the Number of Particles Investigated, and the Summary Statistics for the Three General Classes of Particle Class Averages: Round, Oval and Bilobed

Targeted Complexes	Untilted		RCT		2D Classums			
	CCD Image Count	Particle Count	CCD Image Count	Particle-Pair Count	Total	Bilobed (%)	Oval (%)	Round (%)
Native PS1	300	11,234	52	4,142	200	41	24	35
CpdE-bound PS1	300	10,651	60	990	200	21	29	50

Additional views of ∼200 class averages built in EMAN2 are displayed in Figure S6.

**Figure 6:**
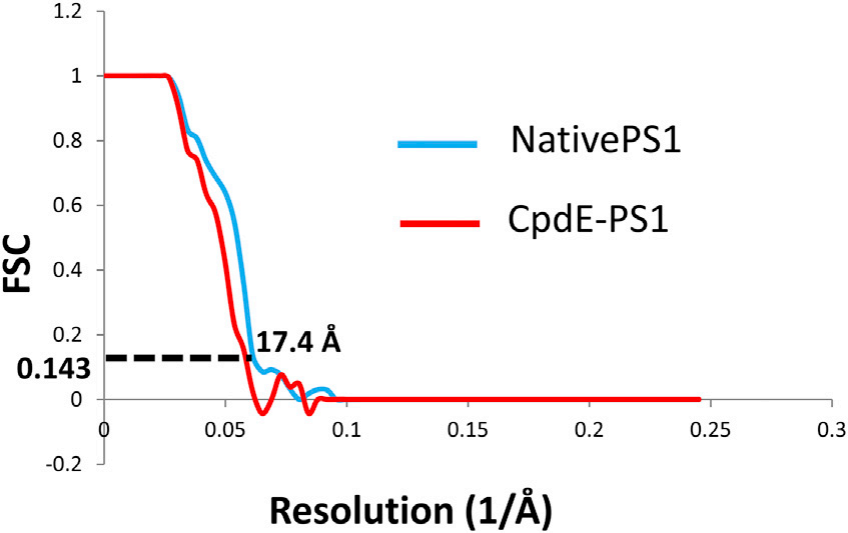
Resolution of the Final Maps as Evaluated by gsFSC Method The 0.143 threshold resolution of both the native and compound E-bound PS1 map was 17.4 Å resolution.

To generate a reliable initial model, we used the random conical tilt (RCT) method for particles with and without compound E (Benefield et al., 2013, Cheng et al., 2006, Guénebaut et al., 1997, Liu and Wang, 2011, Radermacher et al., 1987, Stroupe et al., 2006). Corresponding particles from 0° and 45° tilt pairs were picked using *e2RCTboxer.py* in the Electron Micrograph Analysis software package (EMAN2; 4,142 native particle pairs and 990 compound E particle pairs). The 0° tilt particles were classified using *e2refine2d.py* in EMAN2. The 3D RCT initial model was reconstructed from 45° particles using *e2rct.py*. The initial model created by RCT was initially refined against the RCT particle data set using the Regularised Likelihood Optimisation software package (RELION), filtered to 30 Å using the X-Window-Based Microscopy Image Processing Package (Scheres et al., 2008, Sorzano et al., 2004), and then refined against each full data set (11,234 native PS1 particles and 10,651 compound E particles; Rosenthal and Henderson, 2003) by RELION (Scheres, 2012; Figure S5). The final resolution was calculated using the “gold standard” Fourier shell correlation (gsFSC; Scheres, 2012) within RELION (0.143 threshold resolutions for native PS1 = 17.4 Å; compound E-bound PS1 = 17.4 Å; Figure 6).

### Structure of Native PS1 Complexes

**Figure 7:**
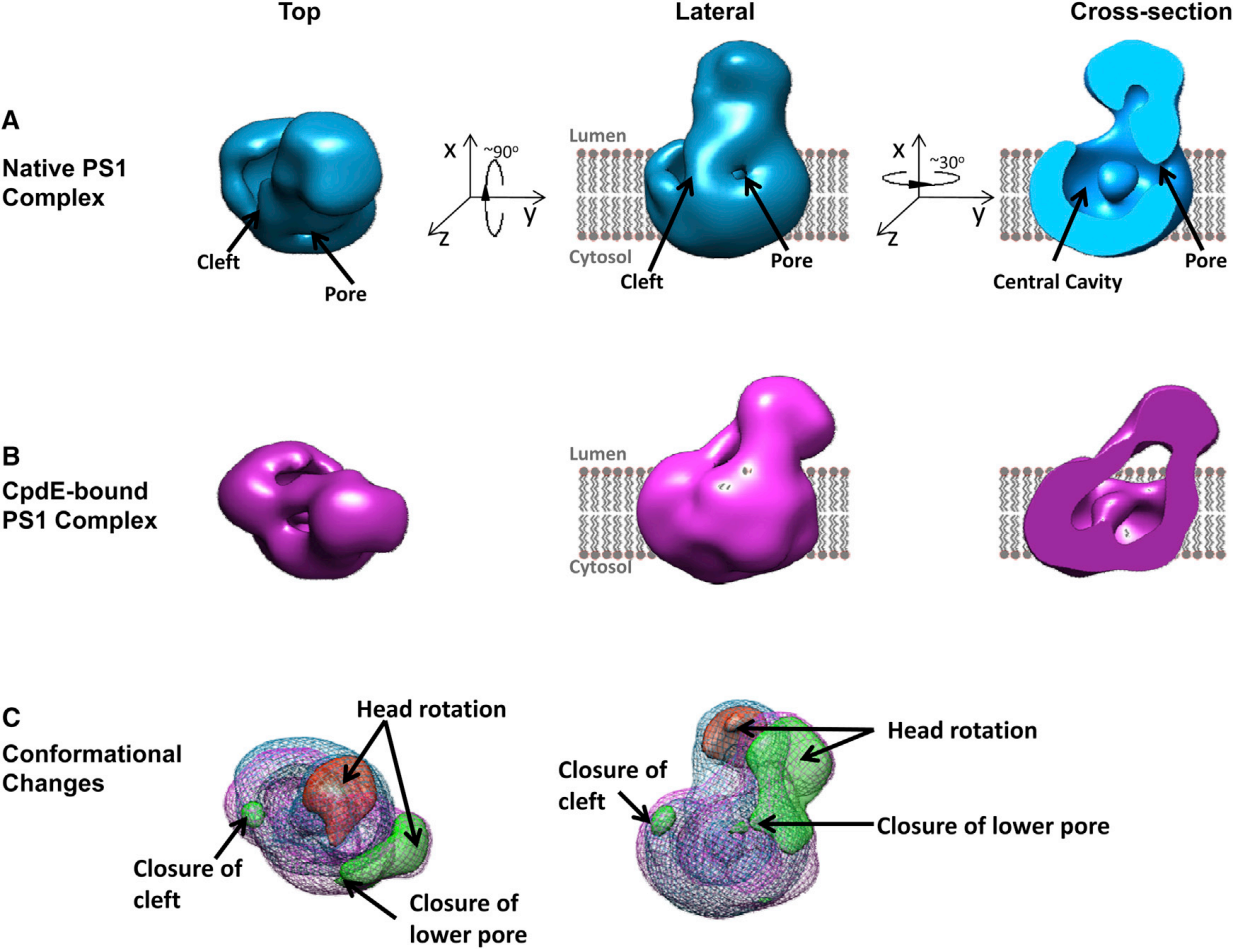
Top-Down Vertical, Lateral, and Cross-Sectional Views of 3D Reconstructions of PS1 Complexes Reveal that Both Native PS1 Complexes and Compound E-Bound Complexes Have an Irregular Bilobed Shape (A) The native PS1 complex contains a head domain and a base domain. The base domain has a lateral cleft and central cavity/channel, which appears to open onto the upper/extracellular surface and also onto the lower surface via a smaller pore. The shaded lipid bilayer represents the boundaries of a putative membrane. (B) 3D reconstructions of compound E-bound PS1 complexes reveal a similar structure, with the rotation and tilting of the head. Density shifts on the external surface of the base result in closure of the lateral cleft and of the lower pore of central channel. (C) Corresponding vertical and lateral views of the difference map, which was calculated using the UCSF Chimera package. Blue mesh is the native PS1 complex, and the pink mesh is compound E-bound PS1. Positive density is represented in green. Negative density is displayed in red. A detailed flowchart of the model-building algorithm is available in Figure S5. A rotating animated video of the native complex built in chimera (Movie S1) and an animated video comparing the native and compound E complexes (Movie S2) are available in online supplemental data files.

The 3D model constructed for native PS1 complexes (EMDB accession number: EMD-2477) had generally similar overall dimensions to those of previously published models (Lazarov et al., 2006, Ogura et al., 2006, Osenkowski et al., 2009, Renzi et al., 2011). However, there were several notable differences. Specifically, the native PS1 complexes had a bilobed conformation rather than the egg-shaped structures of previous models. This bilobed shape had a larger base (93 Å × 93 Å × 60 Å) and a distinct, smaller head (65 Å × 60 Å × 55 Å; Figure 7A).

**Figure 8:**
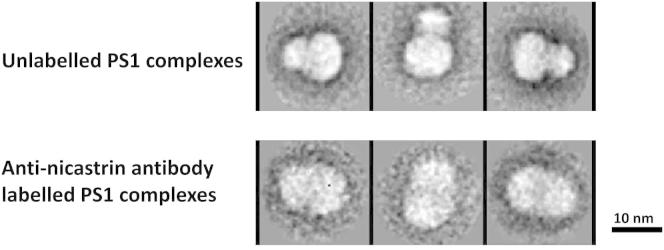
Antibodies to the Ectodomain of Nicastrin Label the Head Domain, Indicating that the Head Domain Is Lumenal/Extracellular Representative class average images of native and anti-nicastrin antibody-labeled PS1 complexes reveal that anti-nicastrin antibody-labeled complexes have an increased density of the head domain. The box width is 261.12 Å.

To establish the orientation of the complex, we exploited the fact that the N terminus of nicastrin is a relatively large (105.9 kDa), heavily glycosylated structure that is known to be located in the lumen/extracellular space. Therefore, we immunolabeled complexes with a monoclonal antibody targeting residues 168–289 in the N terminus of nicastrin. The size of the base domains of these immuno-labeled complexes was not different from that of unlabeled complexes. However, the size of the head domain was considerably larger than that of unlabeled complexes (3,949 particles; Figure 8). Labeling complexes with nonspecific anti-mouse IgGs caused no change in the size of either part of the complex (data not shown). The absence of a single unique location on the head for the increased mass contributed by the anti-nicastrin antibody likely arises from the flexibility of both the antibody and the targeted single-chain ectodomain of nicastrin. At the current resolution, this flexibility in 3D space likely caused the added mass of the antibody to appear as if merged into the mass of the head. The notion that the head contains the nicastrin ectodomain is further supported by the fact that the head of the bilobed structure has a volume of ∼112 nm^3^. This closely approximates the calculated volume (∼128.4 nm^3^) required to contain the known mass of the glycosylated nicastrin ectodomain (105.9 kDa). The other hydrophilic loops in the PS1 complex (residues 1–82 at the PS1–N-terminus, 9.5 kDa; and residues 265–407 in the TM6-TM7 loop, 15.7 kDa) are by themselves too small to account for this structure. Taken together, these observations strongly suggest that the head of the bilobed complex contains the ectodomain of nicastri, and is located in the lumen/extracellular space.

In agreement with a lumenal/extracellular location of the head, several observations suggest that the base is membrane embedded. The height of the base (∼60 Å) is sufficient to span the width of most cellular membranes (35–40 Å; Bondar et al., 2009, Wang et al., 2007) and would be able to contain the TM domains of PS1/PS2, pen2, aph1, and nicastrin.

The membrane-embedded base has a cleft on its lateral surface and contains a series of internal low-density volumes (Figure 7A), which form a central channel that opens onto the lumenal/extracellular surface (Figure 7A). Depending on the z axis rotation of the complex, the lower pore of the central channel may open into the hydrophobic lipid membrane or into the intracellular space (Figure 7A, right panel). Intriguingly, both a lateral cleft between TM6 and TM9 (equivalent of PS1-NTF and PS1-CTF, respectively) and a central channel have been reported in the crystal structure of the Archaeon homolog of the PS1 subunit (MCMJR1; Li et al., 2013).

### Domain Movement Induced by Compound E Binding on PS1 Complexes

To investigate the structural basis for the potent γ-secretase inhibitor activity of compound E, we used the same methods to generate a 3D model of complexes with compound E bound (EMBD accession number: EMD-2478; Figure 6B). We then used two procedures to statistically compare the models.

First, we calculated gsFSC curves (Scheres, 2012) for each model separately. The resultant resolution for both native and compound E-bound PS1 complexes was 17.4 Å (Figure 6), suggesting that the conformational differences observed at this resolution are likely to be reliable.

Second, we calculated a difference map between the two final models. The membrane-embedded base domains of the two models were aligned by “Fit in Map” operation in UCSF Chimera (Pettersen et al., 2004). The models were highly similar, with a correlation of 0.93. The aligned map of the native PS1 complex was then subtracted from that of the compound E-bound PS1 complex, and differences were scored based on thresholding in units of SD. Differences with SD ≥ 7 were displayed as previously described (Figure 7C; Wu et al., 2012). Three significant changes were evident (Figure 7C). First, there were significant mass shifts in the head that effectively rotated and tilted it toward the base. Second, there was significant narrowing or closure of the lower pore in the base of compound E-bound complexes, indicating a more compact structure compared with native complexes (Figures 7B and 7C). Finally, the lateral cleft on the surface of the base appeared to be closed in compound E-bound complexes (Figure 7B).

### Comparison with Prior Models

There are several differences between the current and previous models, which also differed among themselves. The most plausible explanation for these differences is that they arise from the overexpression strategy (often in heterologous nonhuman cell systems) that were employed by all of these previous studies (Lazarov et al., 2006, Osenkowski et al., 2009, Renzi et al., 2011). Such overexpression systems are well known to cause distortions in component stoichiometry of PS1 complexes and incomplete glycosylation of nicastrin (Kimberly et al., 2003). The inclusion of even a small subset of complexes with an abnormal stoichiometry or with incomplete glycosylation of nicastrin would introduce hidden heterogeneity into the sample and cause blurring of the details of the structure.

To circumvent this problem, we deliberately incorporated three features into our purification strategy. First, we used human cells that expressed endogenous, physiologically processed human nicastrin, aph1, and pen2. Second, the only exogenous protein (human PS1) was expressed at near-physiological levels. Finally, we used a multi-step affinity purification protocol designed to eliminate complexes that did not contain both the tagged PS1 and mature nicastrin. We also exploited the higher contrast of negative-stain EM methods which, for small membrane-bound particles with potentially attached lipid, may provide advantages over cryo-EM methods.

### Validation

Single-particle EM of particles of <400 kDa with low symmetry must be interpreted with great care to ensure that 3D models represent the true shapes of the protein particles (see review, Frank, 2006, Frank, 2009, Henderson et al., 2012). We present our data with this caveat in mind. However, three features of our analysis support the notion that the models presented here are probably correct. First, the same bilobed 3D structure was obtained when the model-building process employed an angular reconstruction approach with initial seeding using either bilobed or egg shapes. Second, the same bilobed shape was obtained using an independent data set of particles (n = 3,074 particles). Finally, orientations of the final 3D models built using the RCT-based approach (and two-dimensional [2D] projections of that 3D model) could be found that closely matched both representative raw images (Figure 5A) and reference-free class averages (“classums”) built independently in EMAN2 (Figure 5B).

## Discussion

The experimental results reported here provide four important observations about the structural and functional biology of the presenilin complex.

The first significant outcome of our work is that, by taking advantage of the higher contrast of negative-stain EM methods and by employing an expression/purification protocol that avoids distorting complex stoichiometry, we are able to provide details about the structure of the presenilin complex. Although generally similar in dimensions to previous models (Lazarov et al., 2006, Osenkowski et al., 2009, Renzi et al., 2011), the 3D model presented here has a bilobed shape with distinct head and body domains. The head contains the ectodomain of nicastrin.

Second, in addition to clarifying the general topology of the complex, our work provides further architectural details that were not agreed upon in prior models. In particular, the membrane-embedded base may contain a lateral cleft and a central channel. Similar elements have been observed in the 3.3-Å crystal structure of the Archaeon PS1 subunit homolog. In the Archaeon PS1-subunit homolog, the easily discernible central channel has been interpreted to represent a hydrophobic channel that is distinct from an adjacent shallow solvent-accessible hydrophilic catalytic cavity. However, when the MCMJR1 structure is rendered at 17 Å, the shallow catalytic cavity is not well resolved. Consequently, we are therefore unable to map the corresponding feature on our models.

The distinct cleft between TM6 of the Archeon PS1 subunit (which would be contained in the eukaryotic PS1-NTF hemi-complexes) and TM9 (which would be contained in the eukaryotic PS1-CTF hemi-complexes) has been proposed as a potential initial substrate docking site that might then operate as part of a “lateral gate” mechanism to provide substrate access to the active site (Li et al., 2013). Additional studies will be required to determine whether the lateral cleft observed here in the base of human PS1 complexes also represents the initial substrate docking site. Our pharmacological data provide circumstantial evidence that it may be. Thus, binding of compound E to the complex causes the closure of both the functionally defined initial substrate docking site and the biophysically defined lateral cleft.

The apparent rotation and tilting movement of the nicastrin-containing head in the presence of compound E is of interest. Although controversial, the ectodomain of nicastrin has been proposed to bind the exposed N-terminal stub of substrate proteins after their cleavage by a “sheddase” such as beta-site APP cleaving enzyme (Shah et al., 2005). The observed flexibility of the nicastrin-containing head could facilitate such interactions by bringing the N-terminal ectodomain of nicastrin into closer physical proximity with the N-terminal membrane-bound stub of the substrate.

A third important outcome of the experiments reported here is that they reveal how some non-transition-state γ-secretase inhibitors work. We show that binding of compound E to its binding site on PS1-NTF induces significant allosteric conformational changes in the complex, including closure of the initial substrate docking site. These allosteric effects presumably interfere with the binding and translocation of substrates to the active site. Intriguingly, there is reciprocal crosstalk from the initial substrate docking site to the compound E binding site. Substrate docking increases compound E binding.

It is likely that other small-molecule inhibitors, including the clinically promising class of γ-secretase modulator (GSM) compounds, may work through similar allosteric mechanisms. Indeed, some of the GSMs bind to PS1-NTF (Ohki et al., 2011) and require prior substrate docking for their inhibitor activity (Uemura et al., 2010). Furthermore, these interactions between inhibitor binding sites and initial substrate docking sites are also sometimes substrate specific (e.g., APP but not Notch; Sagi et al., 2011). Additional experiments of the type reported here may help understand the allosteric mechanisms of GSMs at a higher resolution.

Finally, our work suggests that the eukaryotic presenilin complex is likely to be structurally highly dynamic. This structural flexibility might underlie other functionally important long-range interactions within the complex. For example, the operation of the putative “lateral gate,” which governs access of substrate peptides to the catalytic pocket, will likely require reciprocal interactions between the initial substrate binding site and other sites within the complex. These interactions will be required to “open” the gate upon substrate binding and then “close” the gate during peptide translocation to active-site pocket. Our observation of just such reciprocal crosstalk between the initial substrate docking site and the compound E binding site is highly relevant in this regard. Indeed, it is conceivable that the same (or very similar) reciprocal interactions described here between the initial substrate binding site and the compound E binding site are part of this putative “lateral gate” mechanism. Similar long-range dynamic structural effects might also explain how synaptic activity and mutations at diverse locations in the PS1 peptide all affect the relative rates of production of Aβ40 and Aβ42 species (Dolev et al., 2013).

## Experimental Procedures

### Lentiviral Expression of TAP-Tagged Human PS1 in HEK293 Cells

Human PS1 cDNA was tagged at the 5′ end with a TAP tag cassette (Bürckstümmer et al., 2006) composed of Protein G and streptavidin binding peptide tags separated by a tobacco etch virus protease cleavage site. The tagged cDNA was incorporated into a lentiviral vector, transfected into HEK293T cells, and then expressed at near-physiological levels using the WAVE bioreactor system (GE Healthcare).

### Protein Purification

HEK293F cells were harvested at a density of 3 million cells/ml, homogenized in 50 mM Tris-HCl pH 7.4, 150 mM NaCl, 2 mM EDTA, 5 mM MgCl_2_, 5 mM CaCl_2_ at 4°C. Cells were lysed in the same buffer containing protease inhibitor cocktail (Roche), 1% (w/v) digitonin (Calbiochem) for 1 hr, and centrifuged at 100,000 × *g* for 1.5 hr. PS1 complexes were captured on a rabbit IgG-agarose column (Sigma-Aldrich), washed with 100 column volumes of buffer containing 0.04% (w/v) digitonin, cleaved with AcTEV protease (Invitrogen), and eluted with buffer containing 0.04% digitonin. The eluate was purified by Strep-Tactin chromatography (IBA GmbH); eluted in 0.04% digitonin, 5 mM desthiobiotin (Sigma-Aldrich); concentrated on WGA agarose beads (Vector Laboratories); and eluted with buffer containing 0.04% digitonin and 0.5 M *N*-acetyl-D-glucosamine. Protein purity was assessed using NuPAGE Bis-Tris gels (Invitrogen) with silver staining (Pierce). Monodispersity was determined by western blotting of NativePAGE Novex Bis-Tris Gels (Invitrogen) using anti-nicastrin (Sigma N1660) and anti-PS1-NTF (Abcam ab10281) antibodies and compared with NativeMark Unstained Protein Standard (Invitrogen LC0725).

Compound E-bound complexes were purified as above in 0.5–1.0 mM compound E during all steps.

### γ-Secretase Activity Assay

γ-Secretase activity of the PS1 complex was measured by ELISA (Human Aβ40 ELISA Kit; Invitrogen) as described previously (Yu et al., 2000).

### EM

Carbon-coated 400-mesh copper grids were glow discharged in air at 600–700 V for 30–60 s on an Edward S150B sputter coater. Tobacco mosaic virus was mixed with the sample at 0.03 mg/ml. A total of 1.5–3.0 μl of the protein mixture (20 μl/ml) was loaded onto the grid; incubated for 1–2 min; washed five times in 50 mM Tris-HCl pH 7.4, 150 mM NaCl, 2 mM EDTA, 5 mM MgCl_2_, 5 mM CaCl_2_; and blotted on Whatman No. 1 paper. Grids were stained by floating on drops of 1% uranyl acetate for 2–10 s, and the excess of staining reagent was blotted away. The grids were imaged with an FEI Tecnai 12 electron microscope operated at 120 kV. Images were recorded at 67,000× nominal magnification on a 2K × 2K TVIPS 224 charge-coupled device (CCD) camera, resulting in final sampling of 2.04 Å/pixel after correction for the post column magnification.

### 3D Reconstruction of Native and Compound E-Bound Complexes

Particles were picked by *e2boxer.py* (EMAN2) interactively using a square box size of 128 pixels. A total of 11,234 native PS1 particles from 287 CCD images and 9,860 compound E-bound PS1 complex particles from 300 CCD images were picked.

The RCT reconstruction procedure was used for building the reference model. Tilt-pair images for native and compound E-bound PS1 particles were collected as described above, using a 45° tilt angle. A total of 4,142 pairs of native PS1 particles and 990 pairs of compound E-bound particles were picked by EMAN2 (*e2RCTboxer.py*). The 3D reconstruction procedure is as described in Results. Simultaneously, we also used the EMAN2 protocol for generating random initial models (*e2initialmodel.py*) based on the common-lines method. The 2D reference-free alignment and classification of particle projections were performed following EMAN2 routines (*e2refine2d.py*). Particles in each data set (11,234 native PS1 particles and 10,651 compound E particles) were classified to 200 classes using a multivariate-statistical-analysis-based, reference-free classification algorithm. Models calculated using different methods agreed well with each other.

The final 3D model building was as described in Results. Each initial map was refined with the full data set of untilted images (11,234 native PS1 particles and 9,860 compound E particles) by RELION (Scheres, 2012). The resultant resolution, as assessed with the gsFSC method (Scheres, 2012), was 17.4 Å for both native PS1 and compound E-bound PS1 complexes.

The density was then displayed using the UCSF Chimera package (Pettersen et al., 2004), representing a mass of 200 kDa with included volume of 2.45 × 10^5^ Å^3^, assuming a protein density of 1.37 g/cm^3^. This mass is consistent with both the calculated mass of each component protein (215 kDa of PS1 protein complex plus glycosylation) and the protein mass (174 kDa) determined by multi-angle light scattering (Figure 8).

EM-derived density maps have been deposited into the EMDB with EMBD accession numbers: EMD-2477 (native PS1 complex) and EMD-2478 (compound E-bound PS1 complex).

### Immunolabeling

PS1 complexes were immunolabeled by mixing with anti-nicastrin antibody (∼0.1 μM; BD Transduction Laboratories 612290) at a 1:1 molar ratio on ice for 2 hr; applied to 200 μl Strep-Tactin MacroPrep resin pre-equilibrated with buffer containing 0.04% digitonin; mixed for 1 hr at room temperature; briefly centrifuged; and then washed twice in 100 μl buffer containing 0.04% digitonin. Anti-nicastrin antibody-labeled complexes were eluted with 5 mM desthiobiotin, checked by western blotting, immobilized on carbon-coated, glow-discharged copper grids, and imaged.

### Detergent-Induced PS1 Complex Dissociation

Microsomal membranes from native HEK293 cells were pelleted and homogenized in 25 mM HEPES pH 7.4, 1 mM EDTA, 0.25 M sucrose, complete protease inhibitor cocktail (Roche); centrifuged at 3,000 × *g* for 10 min; and the supernatant was then centrifuged at 100,000 × *g* for 1 hr. The microsomal membrane was preincubated with 1% DMSO or 10 pM–100 nM of compound E overnight. Equal amounts of microsomal membranes were solubilized in 25 mM HEPES pH 7.4, 150 mM NaCl, 2 mM EDTA, complete protease inhibitor cocktail with 0.1% (w/v) dodelcyl maltoside (DDM; Affimetrix) for 1 hr. The homogenates were centrifuged at 100,000 × *g* for 30 min, and the supernatant was collected. DDM was added to final DDM concentrations of 0.5%, 1%, and 1.5%. Membrane lysates were incubated overnight at 4°C with anti-nicastrin antibody and Protein G-Sepharose (GE Healthcare). After washing, the captured proteins were eluted with 1× sample buffer (lithium dodecyl sulfate [LDS]; Invitrogen). Samples were resolved on 12% Bis-Tris NuPAGE gels (Invitrogen), transferred onto polyvinylidene fluoride (PVDF) membranes, and probed with anti-nicastrin, PS1-NTF, aph1 (Covance PRB-550P), PS1-CTF (Chemicon MAB5232), and pen2 (Sigma P5622).

### FLIM-FRET

Double-tagged PS1 complexes were generated by placing GFP at the N terminus of human PS1 and by placing RFP carboxy-terminal to the endoproteolysis site in the TM6-TM7 cytoplasmic loop domain (Herl et al., 2006). PS1/2 double-knockout mouse embryonic fibroblasts stably expressing GFP-PS1 (as a control) or GFP-PS1-RFP were solubilized in 1% (w/v) digitonin Tris buffer, enriched with WGA resin, and eluted with 0.5 M *N*-acetyl-D-glucosamine in 0.1% digitonin (w/v) Tris buffer. Protein samples were preincubated overnight with DMSO or 10 μM compound E. The samples were loaded into 0.36-mm-thick borosilicate square capillaries (VitroCom) and sealed with powdered acrylic resin (Lang Dental Manufacturing) with fast curing glue. A SpectraPhysics MaiTai laser (Newport) at 850 nm was used to achieve two-photon excitation of the GFP donor fluorophore. The samples were imaged at 40× with a 515/30 emission filter. Fluorescence lifetime data were acquired using the Becker and Hickl system. The GFP fluorescence lifetimes were fitted to two exponential decay curves and mapped by pseudocolor on a pixel-by-pixel basis over the entire image. Fluorescence lifetimes were converted into FRET efficiency, expressed as follows: FRET efficiency = (t_control_ − t_FRET_)/t_control_ × 100, where t_control_ is the GFP lifetime in the GFP-PS1 construct and t_FRET_ is the GFP lifetime in the GFP-PS1-RFP constructs. Two-tailed unpaired t tests were performed (PRISM version 5; GraphPad), where a p value < 0.05 was considered significant.

### Photo Crosslinking of Noncleavable Substrate

A total of 400–500 μg of microsomal membrane proteins was solubilized in HEPES buffer (25 mM HEPES pH 7.4, 150 mM NaCl, 2 mM EDTA) containing 1% 3-([3-cholamidopropyl]dimethylammonio)-2-hydroxy-1-propanesulfonate and incubated with 10 pM–10 μM compound E. After preclearing with streptavidin-agarose resin (Pierce), membrane lysates were incubated with 100 nM of biotinylated, UV crosslinkable D-helical substrate mimic for 1 hr (Kornilova et al., 2005, Watanabe et al., 2010) and then exposed to 365 nm UV (B100A UV lamp; UV Products) at 7 cm for 40–45 min on ice. Lysates were denatured with 1% nonyl phenoxypolyethoxylethanol, 0.5% sodium deoxycholate, and 0.1% SDS, incubated in streptavidin-agarose resin overnight, and washed. Biotinylated proteins were eluted with 1× LDS sample buffer, resolved on 12% Bis-Tris PAGE gels, transferred onto PVDF membranes, and then probed with anti-PS1-NTF antibodies. Densitometric analysis used ImageJ (version 1.45; National Institutes of Health). Two-tailed unpaired t tests (PRISM, version 5; GraphPad) were used. A p value < 0.05 was considered significant. The reciprocal experiment, in which PS1 was photo-crosslinked with biotinylated, UV-crosslinkable compound E, was carried out as previously described (Fuwa et al., 2007).

### Mass Analysis by SEC-MALS

SEC-MALS was used to determine the mass of the PS1 complex, which was resolved on a Superdex S-200 10/300 analytical SEC column (GE Healthcare) in Tris buffer with 0.1% (w/v) digitonin and detected by UV at 280 nm (Agilent 1200 MWD), light scattering (Wyatt Heleos II), and refractive index (Wyatt Optilab rEX). The masses of the PS1 protein complex and digitonin were determined using the dual detection method as implemented in Wyatt’s ASTRA analysis software as conjugate analysis. The protein refractive index increment used was 0.186 ml g^−1^, and the extinction coefficient for UV detection at 280 nm was 1470 ml g^-1^ cm^-1^ for the PS1 complex. The digitonin refractive index increment was 0.153 ml/g (Burgard, 2009), and the digitonin extinction coefficient for UV detection at 280 nm used was 15 ml/g/cm. The UV value was determined from control measurements of digitonin, injected from a concentrate stock solution in which refractive index (RI) analysis indicated a micelle mass of ∼115 kDa, in agreement with literature values (Burgard, 2009). The UV signal during these measurements was then used to analyze the micelle mass and the UV extinction coefficient was adjusted until a mass consistent with the value determined by RI was obtained. The interdetector delay volumes and associated band broadening constants, as well as the detector intensity normalization constants for the Heleos and the UV intensity calibration, were determined prior to each set of measurements using known protein standards (IgG and BSA).

## Author Contributions

C.-J.T. and Y.L. performed all the single-particle EM and analysis. S.H.-J.L., M.A., O.B., and B.T.H. performed the FRET efficiency analyses. S.H.-J.L., T.T., and T.I. performed the inhibitor and helical peptide binding assays. C.-J.T., C.B., S.Q., R.B.D., and F.C. developed the purification and functional enzyme activity assays. C.M.J. helped with the SEC-MALS study. L.A.F. helped with the statistical analyses. W.M., A.J., and A.M. provided technical assistance. G.S.-U., P.E.F., and P.H.St G.-H. designed the overall research program. All authors contributed to the analysis of the data and writing of the manuscript.

## Accession Numbers

The EMDB accession numbers for the EM-derived density maps reported in this paper are EMD-2477 (native PS1 complex) and EMD-2478 (compound E-bound PS1 complex).

## Supplemental Information

Document S1. Figures S1–S6

Movie S1. Animated Rotating Rendition in Chimera of Native PS1 Complex Showing the Central Cavity, Lateral Cleft, and Potential Lower Pore of the Complex

Movie S2. Animated Rendition in Chimera Morphing between the Native PS1 Complex and the Compound E-Bound PS1 Complex, Showing the Rotation and Flexing of the Head and the Closure of the Lateral Cleft and Putative Lower Pore in the Body
