# Supplementary material for: Structural Interactions between Inhibitor and Substrate Docking Sites Give Insight into Mechanisms of Human PS1 Complexes
Source: Structure. 2014 Jan 7;22(1):125–35. doi: 10.1016/j.str.2013.09.018 (PMC3887256; doi:10.1016/j.str.2013.09.018)

Structure, Volume *21*

Supplemental Information

Structural Interactions between Inhibitor and Substrate Docking Sites Give Insight into Mechanisms of Human PS1 Complexes

Yi Li, Stephen Hsueh-Jeng Lu, Ching-Ju Tsai, Christopher Bohm, Seema Qamar, Roger B. Dodd, William Meadows, Amy Jeon, Adam McLeod, Fusheng Chen, Muriel Arimon, Oksana Berezovska, Bradley T. Hyman, Taisuke Tomita, Takeshi Iwatsubo, Christopher M. Johnson, Lindsay A. Farrer, Gerold Schmitt-Ulms, Paul E. Fraser, and Peter H. St George-Hyslop

SUPPLEMENTAL INFORMATION

Supplemental Figure 1

This figure provides additional information for Figure 2. Construction of a FRET reporter for shifts in PS1 conformation: (A) A GFP tag was placed at the end terminus of PS1, and an RFP tag was placed in the TM6-TM7 hydrophilic loop, such that after complex maturation the PS1-NTF+ pen2 hemi-complex would be labeled with GFP, and the PS1-CTF + aph1 + nicastrin hemi-complex would be labeled with RFP. (B) Western blot of PS1 complexes used in FRET studies: GFP-PS1 (left lane) and GFP-RFP-PS1 (right lane) showing recovery of endoproteolytically-cleaved PS1 after wheat germ agglutinin (WGA) chromatography.

Supplemental Figure 2

Flowchart of TAP purification of PS1 complexes. This supplemental figure supports Figures 5 and 7.

Supplemental Figure 3

Purified PS1 complexes are intact, mono-dispersed and functionally active. (A) Silver stained SDS-PAGE of purified PS1 complex reveals all four core component proteins were present (nicastrin, aph1aph1, pen2, PS1-NTF and PS1-CFT). (B) Blue-Native PAGE of the purified PS1 complex silver-stained or probed by Western blotting reveals only a single species. Left lane was silver stained, central lane was probed with anti-nicastrin antibody, and right lane was probed with anti-PS1-NTF antibody. (C) Activity assay of PS1 complex. Native PS1 complexes were active. The Compound E-bound PS1 complexes were catalytically inactive. This supplemental figure supports Figures 5 and 7.

Supplemental Figure 4

SEC-MALS analysis of intact PS1 complex. The purified PS1 complex was resolved by gel filtration and the intensity of UV absorbance (280nm), light scattering and excess refractive index were determined. The UV and RI values, which are independent measurements of concentration, in combination with the scattered intensity were used to determine the concentration of both the protein and the modifier (in this case, digitonin and any glycosylation since both have similar $\frac{dn}{dc}$ values) by conjugate analysis. The evaluated mass of the PS1 complex was 174 kDa suggesting that the PS1 complex has a 1:1:1:1 stoichiometry in solution. The mass distribution evaluated across the main protein peak was constant indicating that this was a single major species and highly monodisperse. LS is the

intensity of light scattering and RI is the refractive index. This supplemental figure supports Figures 5 and 7.

Supplemental Figure 5

Flowchart for the determination of native PS1 and Compound E-bound PS1 structure models, and comparing the class averages of native PS1 particles to the projections of the final model. This supplemental figure supports Figures 5 and 7.

Supplemental Figure 6

200 class averages generated in EMAN2 for native PS1 complexes (A) and for Compound E-bound complexes (B). This supplemental figure supports Figures 5 and 7.

Supplemental Movie 1

Animated rotating rendition in Chimera of native PS1 complex showing the central cavity, lateral cleft and potential lower pore of the complex. This supplemental movie supports Figure 7.

Supplemental Movie 2

Animated rendition in Chimera morphing between the native PS1 complex (initial) and the Compound E-bound PS1 complex (final), showing the rotation and flexing of the head, and the closure of the lateral cleft and putative lower pore in the body. This supplemental movie supports Figure 7.

Supplemental Figure 1A

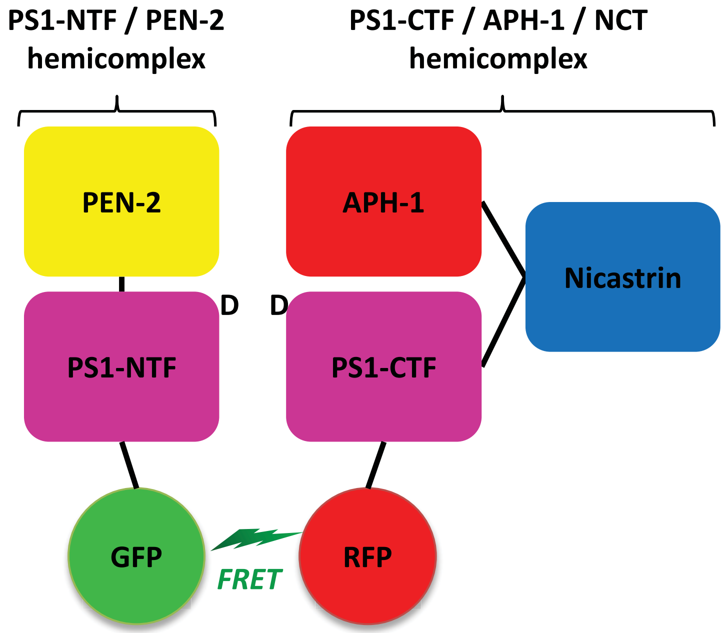

Supplemental Figure 1B

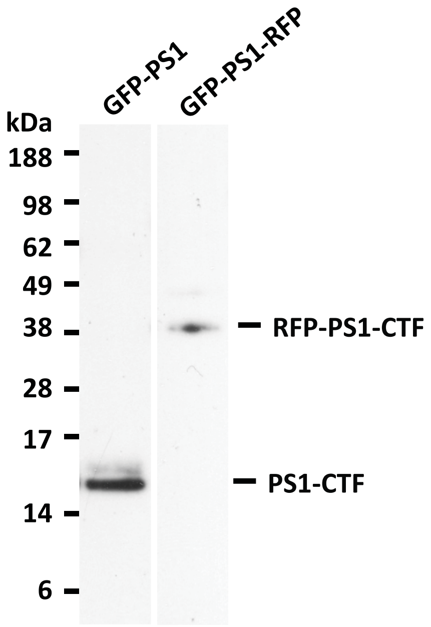

SDS-PAGE Western Blot
Probed with PS1-CTF

Supplemental Figure 2

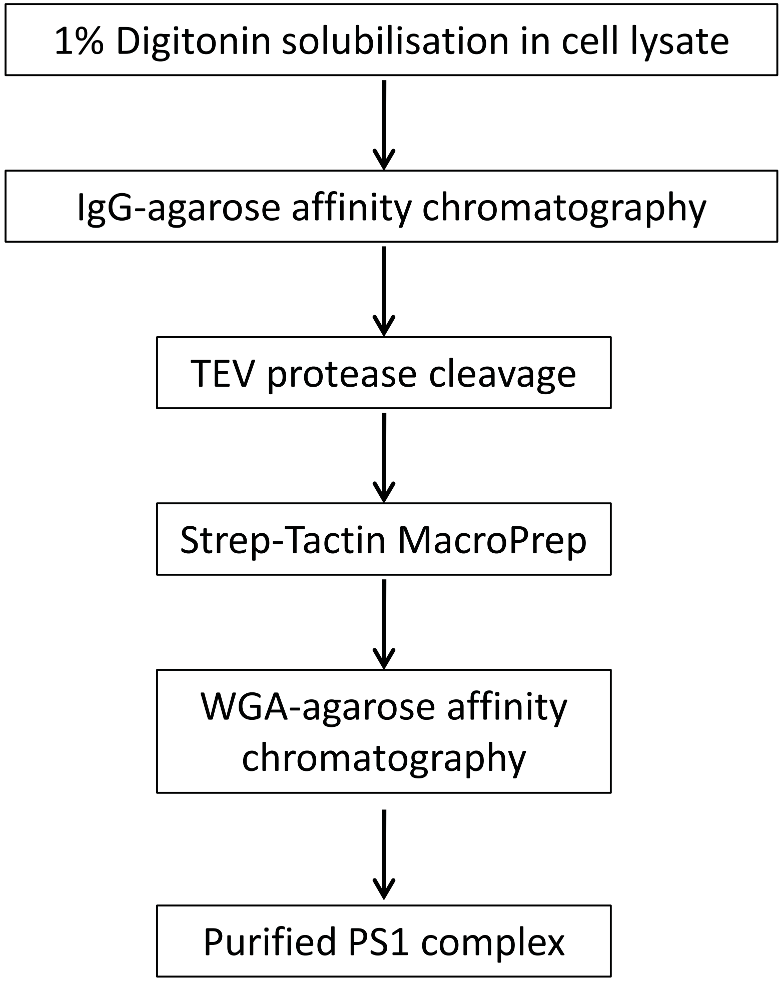

Supplemental Figure 3

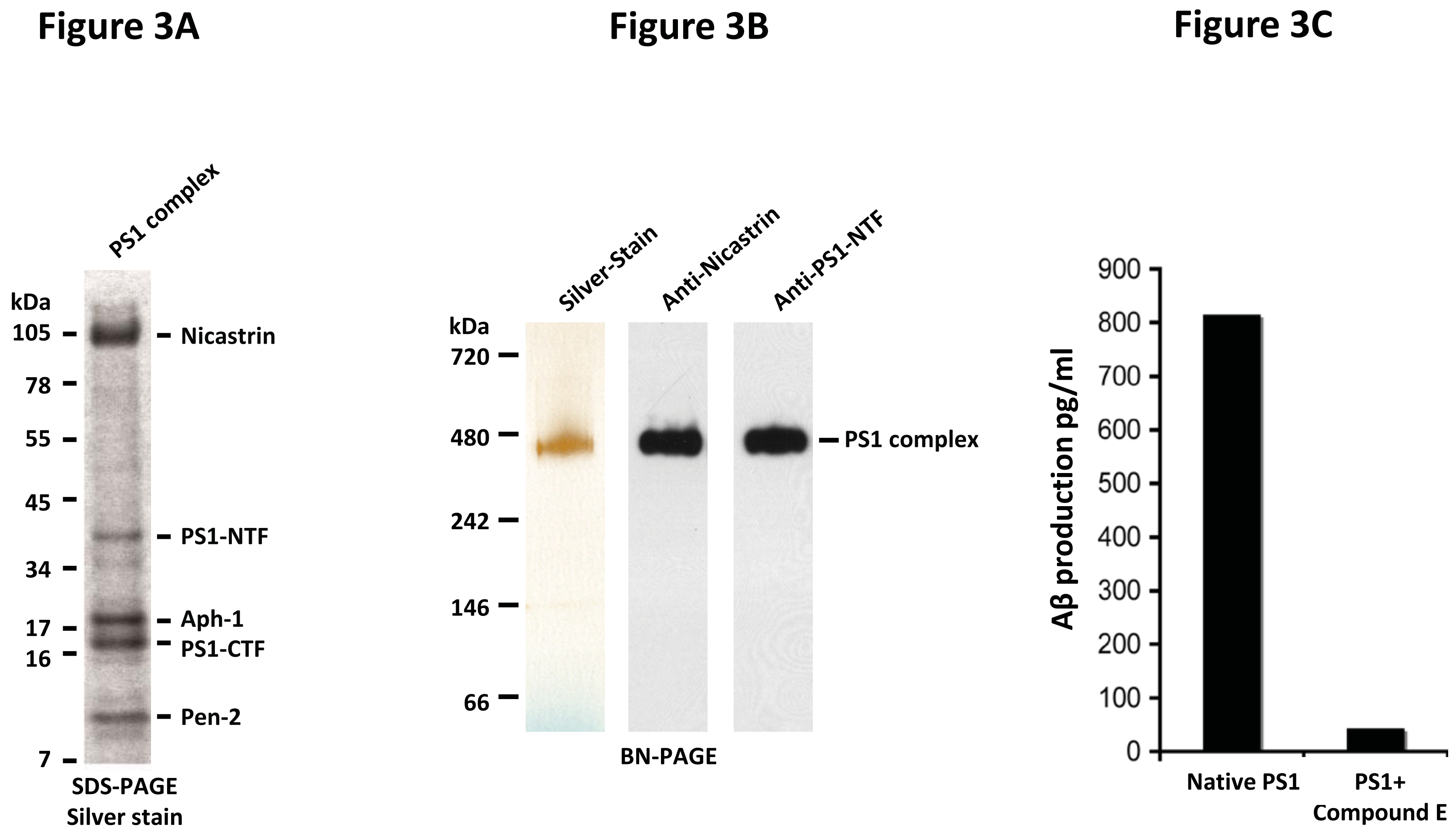

Supplemental Figure 4

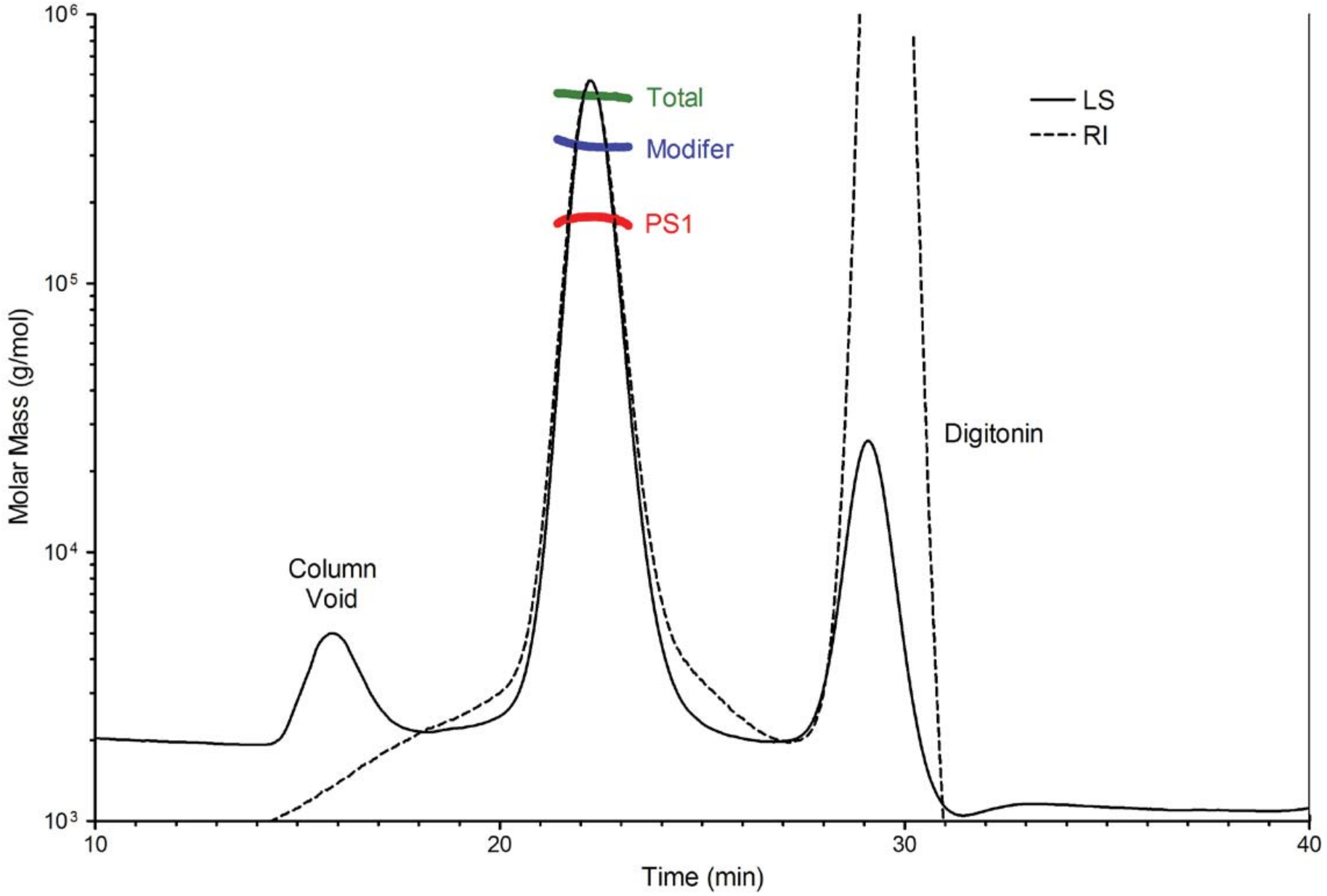

Supplemental Figure 5

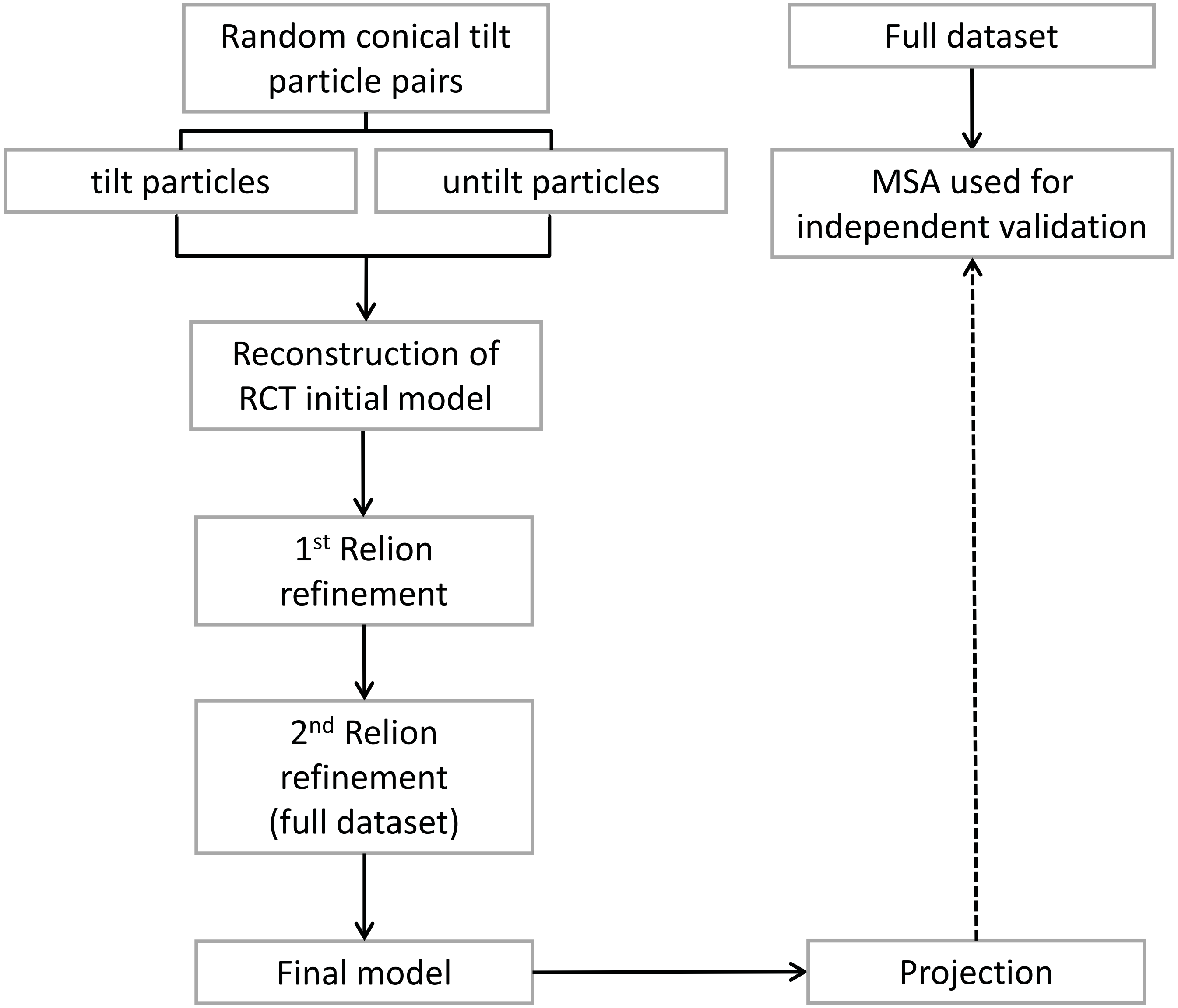

Figure 6A

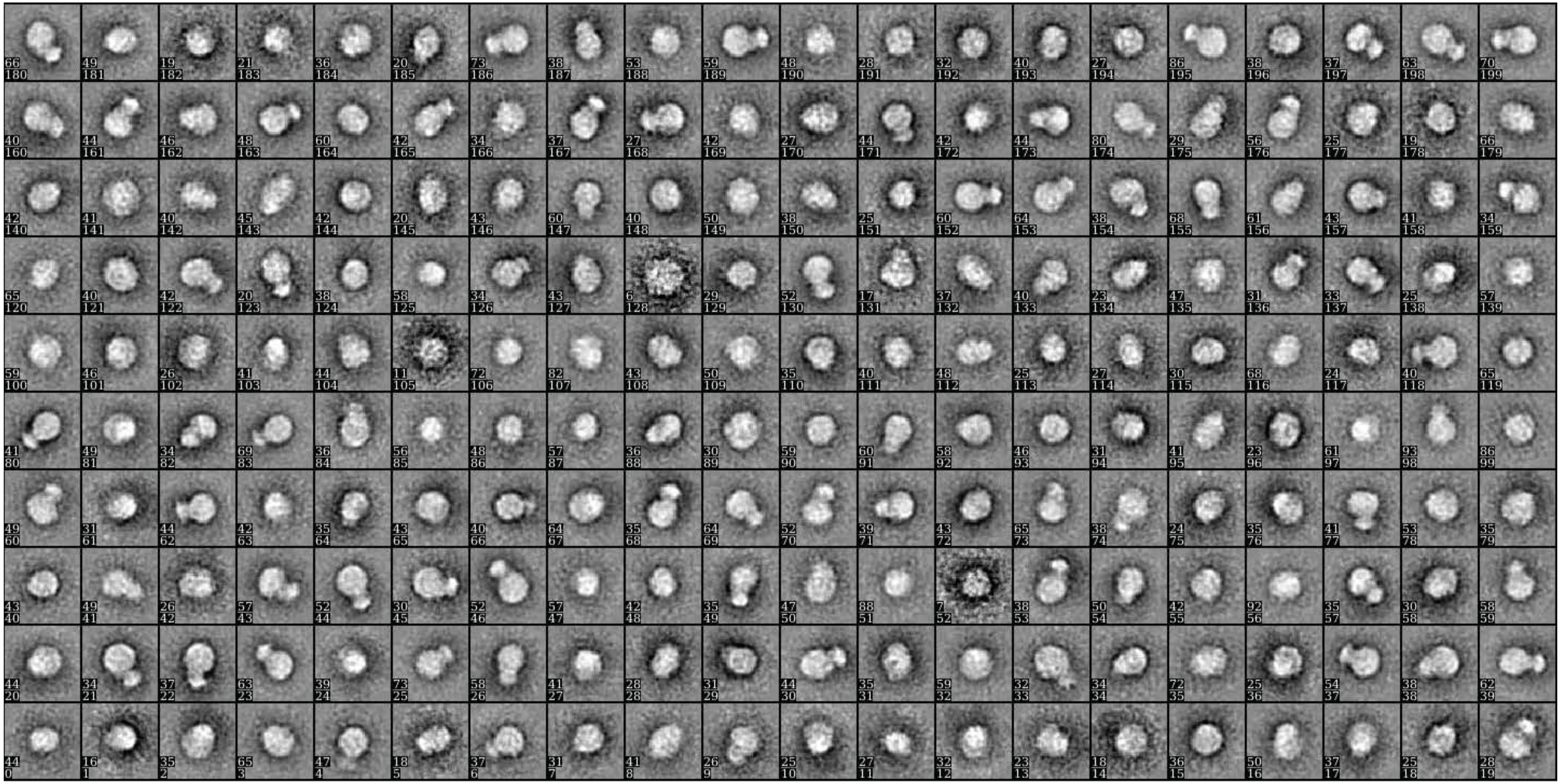

Figure 6B

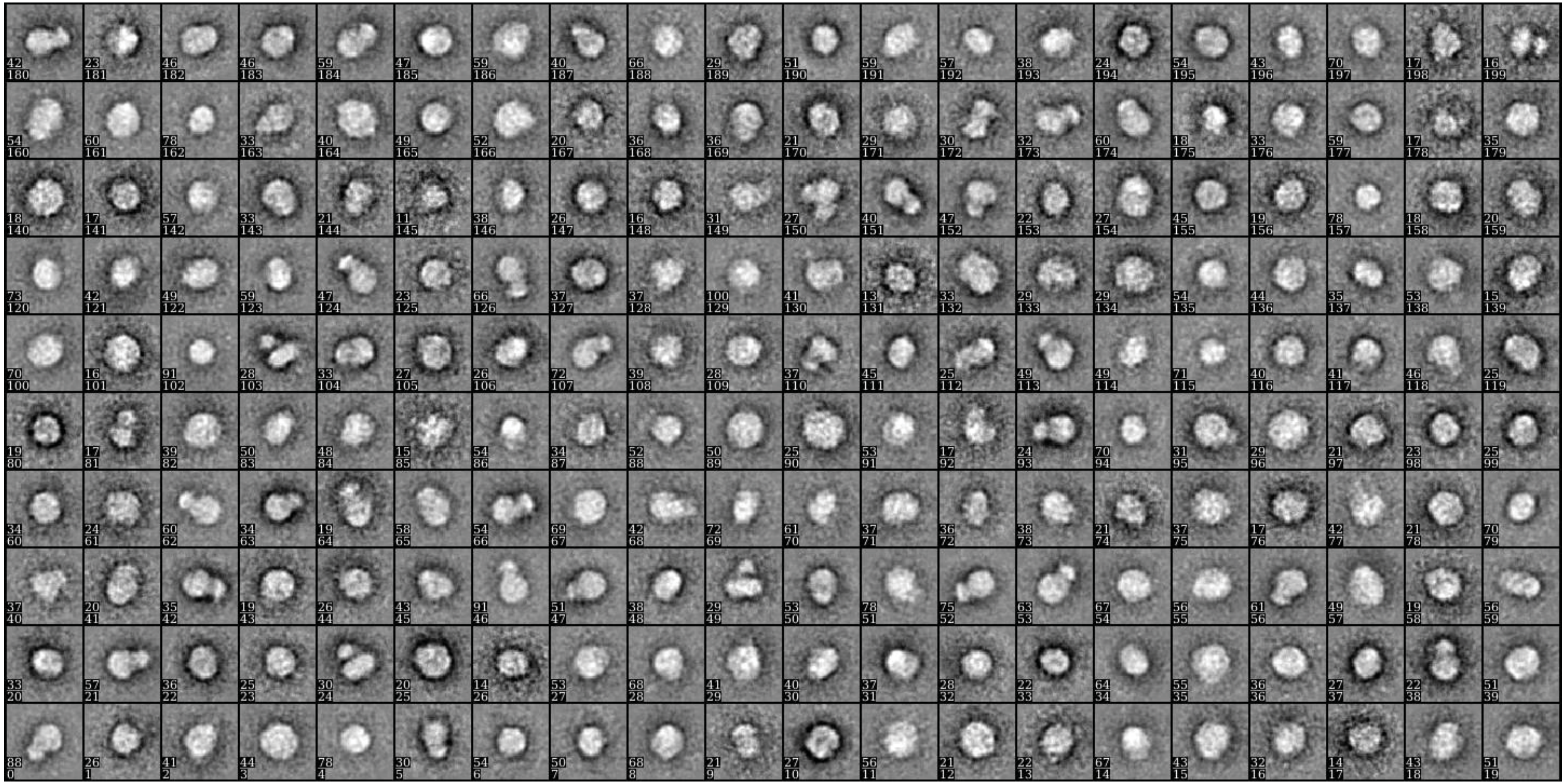

Supplement: Document S1. Figures S1–S6 [file mmc1.pdf]
